# Insomnia Symptoms, Sleep Hygiene, Mental Health, and Academic Performance in Spanish University Students: A Cross-Sectional Study

**DOI:** 10.3390/jcm11071989

**Published:** 2022-04-02

**Authors:** Sara Carrión-Pantoja, Germán Prados, Florian Chouchou, Martha Holguín, Ángela Mendoza-Vinces, Manuela Expósito-Ruiz, Laura Fernández-Puerta

**Affiliations:** 1 Department of Nursing, School of Health Sciences, University of Granada, 18071 Granada, Spain; sara.carrion.pantoja@gmail.com (S.C.-P.); laurafp@correo.ugr.es (L.F.-P.); 2 IRISSE Laboratory (EA4075), UFR SHE, University of La Réunion, 97430 Le Tampon, France; florianchouchou@gmail.com; 3 Department of Nursing, School of Medicine, Catholic University of Santiago de Guayaquil, Guayaquil 090615, Ecuador; mholguinjime@gmail.com (M.H.); amendoza04645@gmail.com (Á.M.-V.); 4 Department of Statistics and Operational Research, University of Granada, 18071 Granada, Spain; mexpositoruiz@ugr.es

**Keywords:** insomnia, sleep hygiene, academic performance, mental health, university students

## Abstract

Background: Insomnia has been associated with decreased academic performance and unhealthy behaviors in university students. Although many studies have analyzed sleep phenomenology among this population, only few have focused on insomnia and its related variables. In addition, such studies do not always include a clinical interview or a specific and validated instrument for measuring insomnia. This study aimed to explore the prevalence of insomnia symptoms and the relationship between insomnia and health habits, mental health, and academic performance in a large university student sample. Methods: Five hundred and eighty-two students were recruited from the University of Granada, Spain. Data were collected through an online survey with questions on sociodemographic and academic data and health habits as well as the Pittsburgh Sleep Quality Index, Insomnia Severity Index, Sleep Hygiene Index, and Sleepiness, Depression, Anxiety, and Stress Scales. A multiple regression analysis explored the relationship between academic performance, health habits, mood state, and insomnia symptoms. Results: The prevalence of students with symptoms of insomnia was high (39.7%). A multiple logistic regression analysis revealed that depression, sleep hygiene, stress and anxiety were significant predictors of insomnia symptoms. Multivariate analyses revealed that subjective insomnia symptoms, sleep efficiency, and depression were significantly correlated with academic performance in a dependent way. Conclusions: In university students, anxiety, stress, and poor sleep hygiene are risk factors for insomnia, which plays an important role in academic performance. Promoting sleep and mental health could be a potentially effective way to improve their academic performance.

## 1. Introduction

University students face a great number of challenges related to their social, personal, and intellectual development. University admission implies major changes for students in their daily habits—including sleep—that can affect their psychological and physical health [1,2]. Regarding the sleep domain, insomnia has a high prevalence among university students (9.4–38.2%) [3,4].

The American Psychiatric Association defines insomnia as difficulty initiating or maintaining sleep, early-morning awakenings, or nonrestorative sleep in adults accompanied by daytime impairments that occur at least three nights per week and last at least three months [5]. In the short term, the consequences of insomnia symptoms are unpleasant body sensations, tiredness, fatigue, and impaired cognition and emotional dysregulation [6,7]. In the long term, the consequences of chronic insomnia have been related to adverse psychological health (e.g., depression, anxiety, risk of suicide) and physical health (e.g., obesity, diabetes, hypertension, cardiovascular disease, poor immune function). In addition, people suffering from insomnia show deficits in cognitive performance, most notably in attention, concentration, and memory [8]. In the student population, insomnia has been associated with decreased academic performance [4,9] and unhealthy behaviors related to food and substance consumption [10,11]. It has also been associated with mental health problems, such as obsessive-compulsive disorders and depression, anxiety symptoms, and suicide [3,12,13].

Although many studies have analyzed sleep phenomenology among this population, only few have focused on insomnia. In addition, such studies do not always include a clinical interview or a specific and validated instrument for measuring insomnia [4,14,15,16]. It is also worth noting that studies focused on insomnia in this population have only considered one or two aspects related to this sleep disturbance (e.g., mental health symptoms [12], suicide [13,17], academic performance [4,18], specific unhealthy behaviors, or sleep hygiene [11,15,19]). As a result, there is a lack of research considering the key variables related to insomnia in this population within a more comprehensive framework.

Poor sleep hygiene involves a wide range of behavioral factors and environmental conditions that may contribute to insomnia [20]. It has been nosologically defined as “inadequate sleep hygiene” by the International Classification of Sleep Disorders [21]. Although many studies have been conducted with university students with the aim of exploring or improving sleep hygiene, very few of them have used specific and validated instruments to measure it. Some of them have included ad hoc questions to assess this nosological entity [22,23]. The Sleep Hygiene Index is the most widespread instrument for measuring these sleep-related behaviors and environmental conditions [24]. As a result, there is a growing number of studies aimed at validating this tool across many countries. Such studies are providing formal scales that make it possible to compare sleep hygiene across studies and guarantee the methodological quality of clinical trials aimed at improving sleep hygiene.

## 2. Material and Methods

### 2.1. Aim

Based on the findings of previous research, the objectives of this study were the following: (1) to assess the prevalence of insomnia symptoms in students as well as their sleep habits and to explore the relationship between insomnia symptoms, sleep hygiene, and mental health in students of the University of Granada (Spain) and (2) to determine the relationship between insomnia symptoms and academic performance of the Spanish student population. We hypothesized that university students reporting insomnia symptoms would have (1) a higher prevalence of mental symptoms and (2) worse academic performance compared to university students not reporting insomnia symptoms.

### 2.2. Study Design and Setting

A cross-sectional observational study was carried out among university students who filled out an anonymous online questionnaire.

### 2.3. Participants and Data Collection

The study sample consisted of 582 students affiliated to various departments: arts and humanities, sciences, social and legal sciences, health sciences, engineering, and architecture (Table 1).

Recruitment was carried out by sending an email to 72 degree coordinators of the University of Granada (Spain) that explained the objectives of the study. If the coordinator accepted to participate, he or she received an email with the explanatory text of the study, an invitation to participate, and the access link to the survey that was to be distributed to the students of the degree through the Official Platform for Teaching Support Resources (PRADO) of the University of Granada. The online questionnaire was administered between January and June 2017. The procedure was approved by the Ethics Committee on Human Research (Reference Code: 312/CEIH/2017), and an informed consent form was sent to participants along with the online survey. Before the students could answer the survey, they had to give electronic informed consent. Students were never asked for any identifying data (e.g., name, ID number).

**Figure 1:**
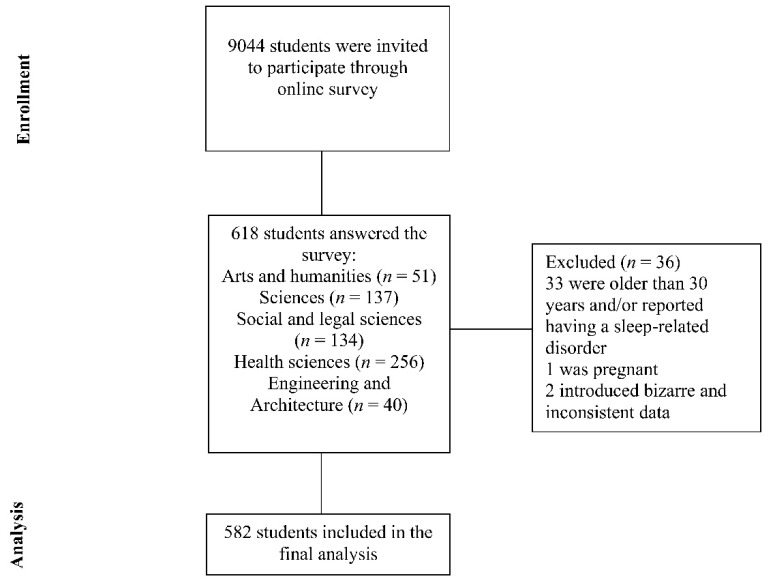
Flow diagram of the research strategy according to the STROBE criteria.

To be included in the study, participants had to be students of the University of Granada and be aged between 18 and 30 years. Exclusion criteria were (1) having a diagnosed sleep disorder other than insomnia, (2) having a severe mental illness in acute phase, and (3) being pregnant. The procedure is presented in Figure 1 according to the STROBE criteria [25]. Of the excluded sample (*n* = 36), 2 participants provided invalid responses to the survey, 1 woman reported being pregnant, 10 participants were older than 30 years, 19 reported having a sleep-related disorder, and 4 reported being older than 30 years and having a sleep disorder (Figure 1).

### 2.4. Measurement Instruments

All the information was collected through the online questionnaire.

#### 2.4.1. Data on Sociodemographic and Academic Aspects and Life Habits

The survey included ad hoc questions in which participants were asked about their age and sex as well as their living, relationship, and economic status. We also collected data related to their lifestyle, that is, weight, height, and consumption of alcohol and tobacco. Level of physical activity was evaluated with the ad hoc question “How many hours per week do you do vigorous or moderate physical activities?”. Students were asked to provide information about their university year and studies. Academic performance was assessed by asking each student to report the current grade point average (GPA) in his/her education record.

#### 2.4.2. Insomnia Severity Index (ISI)

The ISI is a self-reported index that consists of seven items with a 5-point Likert response format ranging from 0 (not at all satisfied) to 4 (very much satisfied). The first three items assess the severity of insomnia; the others measure sleep satisfaction, interference with daytime functioning, perception of the sleep problem by others, and the patient’s level of concern. The total score ranges from 0 to 28, and higher scores indicate greater insomnia severity [26]. The ISI Spanish version has shown an internal consistency of 0.82 in the middle-aged population [27] and 0.91 in the elderly population [28].

#### 2.4.3. Pittsburgh Sleep Quality Index (PSQI)

The PSQI assesses various components of sleep quality, sleep onset latency, sleep duration, sleep efficiency, sleep quality, sleep disturbances, and daytime dysfunction. Each component is assessed on a scale ranging from 0 to 3. The sum of the components provides a score range from 0 to 21. Higher total scores and subscale scores represent worse sleep disturbance. A PSQI global score > 5 denotes clinically problematic sleep [29]. The Spanish version of PSQI has acceptable internal consistency (ranging between 0.67 and 0.81), sensitivity, and specificity [30]. In this study, we considered the global score as well as those of sleep disturbances, sleep efficiency, and sleep-onset latency.

#### 2.4.4. Stanford Sleepiness Scale (SSS)

The SSS was administered as a subjective measure of sleepiness. This instrument has two versions: one measures sleepiness every hour during the day, and the other one assesses the level of sleepiness reported by the participant at three time points of the day (i.e., wake time, middle of the day, and bedtime) in the last week. In this study, we used the latter version. It contains seven statements ranging from 1 (feeling active and vital; alert; wide awake) to 7 (no longer fighting sleep, sleep onset soon; dreamlike thoughts). The total score ranges from to 3 to 21 points. Higher scores indicate a greater level of sleepiness. This scale has shown significant convergent validity with the Epworth Sleepiness Scale and good test-retest reliability [31,32].

#### 2.4.5. Depression, Anxiety, and Stress Scales (DASS-21)

The DASS-21 is a self-reported questionnaire that assesses the severity of the main symptoms of depression, anxiety, and stress in the previous week. The survey consists of 21 statements in a 4-point Likert format ranging from 0 (does not apply to me at all) to 3 (applies a great deal to me most of the time) distributed into 3 subscales (with 7 items each): Depression, Anxiety, and Stress [33]. The Spanish DASS-21 version showed acceptable internal reliability in a sample of university students, exhibiting the Cronbach’s alpha of 0.73, 0.80, and 0.81 for the subscales of anxiety, depression, and stress, respectively [34].

#### 2.4.6. Sleep Hygiene Index (SHI)

The SHI is a 13-item self-reported index that evaluates the presence of behaviors that include sleep hygiene [24]. In each item, participants report how often they perform various specific behaviors (i.e., always, frequently, sometimes, rarely, or never). Higher scores indicate worse sleep hygiene. A translation of the SHI from English into Spanish was carried out for this study. As regards internal consistency, Cronbach’s alpha was 0.7 in the present study.

### 2.5. Data Analysis

SPSS 23.0 software was used for all statistical analyses. The main variables considered were the following: depression, anxiety, and stress—measured with the DASS-21 scales—; sleep hygiene reported by the students—assessed with the SHI—; subjective sleep quality and insomnia symptoms—assessed with the PSQI and ISI, respectively—; and academic performance—based on participants’ grade point average. Other secondary variables considered were sociodemographic and academic characteristics, sex, age, weekly physical activity, body mass index (BMI), and alcohol and tobacco consumption.

The main dependent variable was insomnia severity assessed with the ISI. The sample was divided into two groups according to insomnia symptoms. The cut-off point was established through the scores obtained in the ISI. A score of 10 was considered to reflect an insomnia complaint even though it is below the clinical threshold [26]. According to Morin et al., a cut-off score of 10 in the ISI appears to be the best compromise to achieve optimal balance between sensitivity and specificity in a population-based sample [35]. The Kolmogorov–Smirnov test was used to test for normality of the continuous variables. Non-parametric tests were performed because some variables were not normally distributed. Descriptive statistics (i.e., median (interquartile range) and/or percentages) were used to describe the characteristics of the study sample. Categorical variables were analyzed with Pearson’s chi-square test and Fisher’s exact test, where appropriate. Mann–Whitney tests for non-parametric distributions (for continuous variables) were used for comparisons between groups.

To determine the relationship between sleep perception and health habits, separate univariate and multivariate logistic regression analyses were performed between the insomnia variable (measured with the ISI) and the variables that were significantly different between both groups. A logistic regression analysis was carried out to build predictive models of insomnia and a backward stepwise selection method was applied (considering insomnia as a dichotomous variable). Variables with a *p* < 0.05 value in the bivariate analysis were introduced into the model.

Finally, to determine the relationship between academic performance and sleep, sleep hygiene, and mental health, separate univariate and multivariate regression analyses for continuous variables were performed between the GPA, PSQI, SHI, ISI, and DASS-21 questionnaires. Adjustments in multivariate models were made for all variables that were significantly associated with the GPA in the univariate analysis.

## 3. Results

### 3.1. Description of the Sample

The flow diagram in our study included 9044 students who received the online survey from 22 coordinators degree through the official platform of the university (Figure 1). Of a total of 618 of students who completed the online survey, 582 met the inclusion criteria and were included in the final data analysis. The median age of participants was 21 (19–22), and 357 were women (63.3%). Most students were single (94.5%) and lived with roommates (51.4%) or with their parents or guardians (34.9%). More than two-thirds (69.9%) reported a household income that did not exceed EUR 2000, and 24.6% reported that a household income below EUR 1000. Most students were equally distributed among the four years of the studies (first year, 26.3%; second year, 24.7%; third year, 21.5%; fourth year, 23.2%; and others, 4.3%), and 47.1% of them were in the exam period when they answered the survey. In addition, 42.1% of students were enrolled in health-related degrees.

### 3.2. Insomnia Complaints and Sociodemographic-, Lifestyle-, Academic-, and Psychological-Related Factors

Considering the epidemiological cut-off score of the ISI chosen for this study, 232 (39.7%) of the 582 students of the sample had significant insomnia complaints (ISI total score > 10). According to the clinical classification of insomnia severity measured by the ISI, 15.8% of the students reported mild clinical insomnia, and only 1.5% reported severe clinical symptoms of insomnia. Table 1 summarizes the characteristics of the students divided according to ISI scores (ISI total score > 10). There were no significant differences in sex, relationship status, living status, or household income between these two groups.

Scores concerning perceived sleep quality measured by the PSQI were consistent with the two groups established according to insomnia features. Specifically, students with insomnia reported worse global sleep quality (8 (5–10) vs. (6 (4–7)), more sleep disturbances, lower sleep efficiency, and higher sleep latency than students without insomnia (for all, *p* < 0.001).

Regarding lifestyle habits, students performed 4 (2–6) hours of physical activity per week and the BMI was 22.5 (20.4–24.8). Only 19.7% and 14.9% of the students, respectively, consumed alcohol and tobacco on a regular basis. There were no significant differences in these variables either between students who reported having insomnia and students who did not.

Significant differences were observed in the DASS-21 scores, with higher depression (5 (2–10)), anxiety (5 (2–8)), and stress (9 (5–12)) values in students with insomnia complaints compared to normal sleepers. Moreover, higher scores in the SHI showed that students reporting insomnia had significantly worse sleep habits. The difference in sleep hygiene was also significant, with higher scores in the SHI (20 (16–25)) in students with insomnia symptoms. Finally, students with insomnia reported higher sleepiness than students without insomnia (Table 1).

Regarding academic aspects (Table 2), 274 students were in the exam period, and 52.2% of them reported insomnia complaints. Thus, being in the exam period was a significant factor for reporting insomnia symptoms (*p* = 0.046). There were no differences in insomnia complaints between students in the various university degrees, but a significant relationship was observed in students who were enrolled in a health-related degree. Specifically, only 34.5% of students with symptoms of insomnia were enrolled in a health-related degree compared to 65.5% who were not studying for a health-related degree. As regards academic performance, significant differences were found (*p* < 0.001) between students with and without insomnia complaints, with a higher GPA in students who did not report insomnia symptoms.

### 3.3. Predictors of Insomnia Symptoms

As part of our first objective in this study, we performed a logistic univariate and multivariate regression analyses to assess predictors of insomnia symptoms in university students. Continuous and categorical variables that were significant in the statistical group comparisons were assessed as predictors in the analysis (i.e., exam period, health science-related studies, insomnia, depression, anxiety, stress, and sleep hygiene). In the crude model, we found the following significant predictors of insomnia symptoms: depression, with an OR of 1.16 (1.12–1.21), anxiety 1.10 (1.02–1.18), stress 1.10 (104–1.17), and sleep hygiene 1.08 (1.05–1.12). The predictive capacity of the model was high, with an area under the curve of 0.776 (see Table 3).

Finally, adjusting for university year and exam period as covariates, the multivariate analysis showed that anxiety, stress, depression, and sleep hygiene were also predictors of insomnia symptoms (for all, *p* < 0.05). A second model including all these significant variables revealed that stress and sleep hygiene were the strongest significant independent predictors of insomnia.

### 3.4. Predictors of Academic Performance

The second objective in this study was carried out by using univariate regression analyses to assess predictors of academic performance among university students. The analyses revealed a relationship between the GPA and subjective sleep quality measured by the PSQI (i.e., total score, sleep disturbances, sleep efficiency, and sleep latency), sleep hygiene, insomnia severity, and depression, anxiety, and stress (Table 4). A first multivariate model including sex, age, BMI, university year, exam period, physical activity, and alcohol and tobacco consumption as covariates showed that sleep efficiency measured by the PSQI, ISI scores, and depression were related to academic performance (for all, *p* < 0.05). A final statistical model including all the variables previously found to be significant failed to show significant relationships, showing that such variables influence academic performance in a mutually dependent way.

## 4. Discussion

The present study explored the prevalence of insomnia symptoms and its relationship with mental health, academic performance, and health habits in a sample of Spanish university students. We observed the following: (1) The university student population showed a high prevalence of insomnia symptoms with clinical and non-clinical thresholds (17.3% and 37.5%, respectively); students with insomnia reported higher levels of depression, anxiety, stress, and sleepiness and poorer sleep hygiene than students without insomnia. Moreover, a multivariate regression analysis showed that stress and sleep hygiene were independent predictors of insomnia among university students. (2) Insomnia severity, sleep efficiency, and depression symptoms were associated with decreased academic performance in a dependent way.

### 4.1. High Prevalence of Insomnia Symptoms in University Students

The prevalence of insomnia symptoms found in the present study is consistent with previous studies that have analyzed insomnia in the student population using the ISI [12,19] and the Diagnostic and Statistical Manual of Mental Disorders (DSM) criteria for chronic insomnia [36]. More broadly, a systematic review showed a prevalence of insomnia ranging between 9% and 38% in university students [14]. Although the heterogeneity of the tools and thresholds used does not make it possible to accurately determine the prevalence of insomnia among students, it is higher than that reported in the general population. A recent international epidemiological study has reported a prevalence of insomnia of about 11% of the general population in European countries [37]. Taken together, these studies as well as and ours highlight that university students have difficulties sleeping well. Several reasons have been proposed, such as stress concerning their future life and employment, late-night computer work, and social interaction or environmental noise [1,38]. In our study, reported stress and sleep hygiene appeared as two strong independent factors contributing to sleep disruptions regardless of their origin.

### 4.2. Relationship between Insomnia Symptoms, Depression, Anxiety, and Stress

Some studies have found a relationship between insomnia and mental health symptoms in university students. Specifically, insomnia has been associated with an increase in depression, anxiety, and stress symptoms [3,12,36,39]. Similar to these previous studies, university students who reported insomnia complaints in the present research exhibited higher levels in these three psychological variables measured by the DASS-21. Additionally, a multivariate analysis showed that stress was a significant independent predictor of insomnia. In line with our findings, two studies have shown a significant and direct effect of perceived stress on sleep difficulties [40,41]. Although stress, depression, and anxiety have been described as insomnia-related consequences, there is evidence that insomnia is bidirectionally related to anxiety and depression in general population [42]. The relationship between insomnia an emotional status has also been associated with unhealthy behaviors in university students. In fact, Younes and colleagues found in a sample of medical students that potential Internet addiction was related to higher scores in insomnia, depression, and stress as well as lower self-esteem [43]. Concerning food patterns, Arbues and colleagues found that excessive intake of sweets and low intake of dairy products were associated with a higher prevalence of insomnia, stress, anxiety, and depression [10]. Regarding this relationship between insomnia, emotional status, and unhealthy behaviors in university students, it is worth mentioning the following: Kauffman et al. made a theoretical observation pointing out the existence of a transdiagnostic mechanism in the relationship between insomnia, emotional dysregulation, and problematic food behaviors among students [15]. Along these lines, research in the clinical population has suggested that insomnia and emotion dysregulation may act as potential transdiagnostic processes that are relevant across a range of psychopathology symptoms [44].

### 4.3. Relationship between Insomnia Symptoms and Sleep Hygiene

In the last few years, the concept of sleep hygiene has become more and more recognized. Practices such as keeping the bedroom too brightly lit or too noisy, nighttime alcohol consumption, smoking, and use of other substances that can interfere with sleep are common among university students [1]. In our sample, students of the University of Granada with higher insomnia levels had worse sleep hygiene. This finding is consistent with those of a study carried out by Gellis and colleagues [19]. These researchers found that students who reported higher levels of insomnia had worse sleep schedules and worse behaviors close to bedtime and also slept in more uncomfortable environments. In keeping with these results, sleep hygiene was identified as one of the predictors of insomnia [45]. Peach and colleagues also demonstrated that poor sleep hygiene has both direct and indirect effects on subjective well-being and depression in university students [46]. In clinical trials with university students, providing sleep hygiene education has shown small effect sizes regarding sleep and sleep-related variables; by contrast, cognitive-behavioral therapy (CBT) focused on insomnia with sleep hygiene as a component has yielded large effects concerning all sleep variables and dysfunctional beliefs about sleep [22]. Although sleep hygiene is a key component of the psychological treatment of insomnia in adults, evidence to date does not support its use as a stand-alone therapy for insomnia [47].

As stated before, in our study, being in the exam period was related to higher levels of insomnia complaints. Our findings agree with a previous study that reported the presence of symptoms of stress and sleep disturbances among university students in the pre-exam period [48,49]. The cross-sectional nature of the present study did not allow us to explore within-person variations in insomnia scores between the different periods of the academic year (i.e., non-exam period vs. exam period). It should also be noted that the operationalization of the “sleep period” variable in our research included pre-exam periods and exam periods. This refers to established three-week-long academic periods in the university where the recruitment took place, when students prepare for and take examinations. There are no lectures during these exam periods. Moreover, the recruitment process of this research took place from January to June. This period covered non-exam periods but also two pre-exam and exam periods and the preparation for extraordinary exams held at the beginning of July. All this partly explains the distribution of the sample (non-exam period: 52.9% vs. exam period: 47.1%). It makes it difficult to compare our study with those that have explored the influence of exam periods on students’ sleep-related outcomes establishing more specific categories [50].

Academic stress is related to students’ bad sleep habits [51]. However, there is some evidence that health science students adopt more health-related behaviors compared to non-health science university students [52]. The findings of the present study agree with the idea that students of health sciences may develop better sleep habits based on their training and awareness about the importance of sleep in health.

The findings of the present study suggest that is important to assess both insomnia and emotional status in university students through educational university programs and refer and treat students who are clinically affected. It is worth noting the interplay between insomnia, depression, and anxiety [3,12,36,39] and the existence of dysfunctional cognitive and emotional processes besides inappropriate sleep behaviors [1,40,53]. For these reasons, the use of cognitive-behavioral therapy focused on insomnia appears to be the most appropriate treatment according to previous clinical trials focusing on sleep disturbances in this population [22]. In addition, some components of this therapy, such as sleep hygiene and psychoeducation about sleep, should be adapted and used in programs addressing health-related outcomes and healthy lifestyle behaviors during university life.

### 4.4. Insomnia Symptoms as a Predictor of Academic Performance

As regards academic performance, several studies have shown that sleep loss, poor sleep quality, irregular sleep/wake rhythms, and insomnia influence this variable in university students [18].

Specifically, Phillips et al. found in 61 undergraduate students that irregular sleep and light-exposure patterns were associated with delayed circadian rhythms and lower academic performance [54]. A study conducted in the United States showed that students with no sleep disorders had a higher GPA than did those who reported at least one sleep disorder [55]. A recent study carried out among Norwegian higher education students reported that insomnia was associated with a high risk of failing tests [4]. The findings of these studies are in line with those of the present study, in which a significant relationship between insomnia and GPA was observed. This relationship could be explained by the consequences of insomnia, which can hamper the learning capacity of the students due to impairments in memory consolidation and some aspects of executive functioning, such as diurnal attention [56]. Altered sleep habits in university students causing sleep deprivation, disrupted sleep, and irregular sleep/wake patterns can also reduce the intricate interplay between slow-wave activity and other brain rhythms occurring during non-rapid eye movement sleep. This neurophysiological activity is associated with memory consolidation and some aspects of executive functions that involve attention, fluency, reasoning, and switching [57].

### 4.5. Limitations and Perspectives

This study has several limitations. First, because of its cross-sectional design, it was not possible to establish a causal relationship between the study variables that could be of interest for future research. Second, we did not use an interuniversity representative design, and the response rate of our survey was low, which may hamper the representativeness and generalizability of our results concerning Spanish university students and students in our university. Third, we relied on online self-reported measures and were unable to include personal interviews to make an accurate diagnosis of insomnia in each student. As a result, the measures obtained by the ISI may have overestimated the prevalence of clinical symptoms of insomnia.

These findings suggest the need to conduct further studies of sleep in the student population with larger samples. This would be useful to determine more precisely how the variables analyzed in this study are interrelated and to be able to generalize the findings beyond the context of the University of Granada. Moreover, longitudinal studies could also contribute to determine the sensitivity or bidirectionality between these variables.

## 5. Conclusions

In summary, this study suggests that insomnia symptoms play an important role in students’ academic success and mental health. Stress and its management as well as sleep hygiene are two independent components related to insomnia symptoms reported by students. Moreover, sleep efficiency and the severity of insomnia symptoms reported by students may in turn contribute to academic performance through psychological aspects such as certain depression symptoms. Some university innovation projects and action plans for students should envisage research in this field of student health, developing psychoeducational strategies for the improvement of sleep during student life and offering psychological interventions to students with clinical symptoms.

## Figures and Tables

**Table 1:** Demographic data, health habits, and mental symptoms of the sample according to insomnia symptoms.

	Total (*n* = 582)	No Insomnia Symptoms (*n* = 350)	Insomnia Symptoms (*n* = 232)	*p*
Sex (*n*, %)				0.640
Male	225 (38.6%)	138 (39.4%)	87 (37.5%)	
Female	357 (63.3%)	212 (60.6%)	145 (62.5%)	
Relationship status (*n*, %)				0.779
Single	550 (94.5%)	330 (94.3%)	220 (94.8%)	
Committed relationship †	32 (5.5%)	20 (5.7%)	12 (5.2%)	
Living situation (*n*, %)				0.606
Parent/guardian	203 (34.9%)	129 (36.9%)	74 (31.9%)	
Roommates	299 (51.4%)	174 (49.7%)	125 (53.9%)	
Alone	31 (5.3%)	17 (4.9%)	14 (6.0%)	
Other	49 (8.4%)	30 (8.6%)	19 (8.2%)	
Monthly household income (*n*, %)				0.068
Less than EUR 1000	143 (24.6%)	76 (21.7%)	67 (28.9%)	
From EUR 1000 to EUR 2000	264 (45.3%)	157 (44.9%)	107 (46.1%)	
From EUR 2000 to EUR 3000	120 (20.6%)	83 (23.7%)	37 (15.9%)	
More than EUR 3000	55 (9.4%)	34 (9.7%)	21 (9.1%)	
Significant alcohol consumption (*n*, %)				0.377
No significant alcohol consumption	467 (80.2%)	285 (81.4%)	182 (78.4%)	
Significant alcohol consumption	115 (19.7%)	65 (18.6%)	50 (21.6%)	
Tobacco consumption (*n*, %)	87 (14.9%)	45 (12.9%)	42 (18.1%)	0.082
Age (y)	21 (19–22)	21 (19–22)	21 (19–22)	0.521
Body mass index (kg/m^2^)	22.5 (20.4–24.8)	22.4 (20.4–25.0)	22.5 (20.4–24.7)	0.593
Hours exercise/week (h)	4 (2–6)	4 (2–6)	4 (1.5–6)	0.139
ISI-Insomnia (/28)	9 (6–13)	7 (4–8.25)	14 (12–16)	< 0.001
PSQI total (/21)	7 (4–9)	6 (4–7)	8 (5–10)	< 0.001
PSQI disturbances (/3)	1 (1–1)	1 (1–1)	1 (1–2)	< 0.001
PSQI efficiency (/3)	1 (0–1)	0 (0–1)	1 (0–1)	< 0.001
PSQI latency (/3)	1 (1–2)	1 (1–2)	2 (1–2)	< 0.001
DASS-Depression (/7)	3 (1–7)	2 (1–4.25)	5 (2–10)	< 0.001
DASS-Anxiety (/7)	3 (1–6)	2 (1–4)	5 (2–8)	< 0.001
DASS-Stress (/7)	6 (2–10)	4 (1–7)	9 (5–12)	< 0.001
Sleep Hygiene Index (/52)	17 (13–22)	15 (11–19.25)	20 (16–25)	< 0.001
Stanford Sleepiness Scale (/24)	10 (8–12)	9 (7–11)	11 (9–13)	< 0.001

Values are presented as median (interquartile range) or number (percentage) when appropriate. DASS, Depression, Anxiety, and Stress Scale; ISI, Insomnia Severity Index; PSQI, Pittsburgh Sleep Quality Index. † includes engaged, married, and in a domestic partnership.

**Table 2:** Academic data of the sample according to insomnia symptoms.

	Total (*n* = 582)	No Insomnia Symptoms (*n* = 350)	Insomnia Symptoms (*n* = 232)	*p*
Exam period (*n*, %)				0.046
Non-exam	308 (52.9%)	197 (54.3%)	111 (47.8%)	
Exam	274 (47.1%)	153 (43.7%)	121 (52.2%)	
University year (*n*, %)				0.746
Freshmen students	153 (26.3%)	86 (24.6%)	67 (43,8%)	
Sophomore students	144 (24.7%)	90 (25.7%)	54 (37,5%)	
Junior students	125 (21.5%)	72 (20.6%)	53 (42,4%)	
Senior students	135 (23.2%)	87 (24.9%)	48 (35,6%)	
Fifth-/Sixth-year students	20 (3.4%)	12 (3.4%)	8 (3.4%)	
Master’s/Other students	5 (0.9%)	3 (0.9%)	2 (0.9%)	
Health Sciences (*n*, %)				0.002
Non-health-related degree	337 (57.9%)	185 (52.9%)	152 (65.5%)	
Health-related degree	245 (42.1%)	165 (47.1%)	80 (34.5%)	
GPA	7.2 (6.6–8.0)	7.5 (6.7–8.0)	7.0 (6.4–7.9)	< 0.001

Values are presented as median (interquartile range) or number (percentage) when appropriate. GPA, grade point average.

**Table 3:** Odds ratio (OR) for variables predicting status of insomnia symptoms.

	Crude OR	CI 95%	*p*	Adjusted OR 1 †	CI 95%	*p*	Adjusted OR 2 ‡	CI 95%	*p*
DASS_Depression	1.16	1.12–1.21	< 0.001	1.16	1.11–1.22	< 0.001	1.02	0.96–1.09	0.470
DASS_Anxiety	1.10	1.02–1.18	0.010	1.24	1.18–1.32	< 0.001	1.07	0.99–1.12	0.092
DASS_Stress	1.10	1.04–1.17	< 0.001	1.20	1.15–1.25	< 0.001	1.10	1.03–1.18	0.003
SHI_total	1.08	1.05–1.12	< 0.001	1.12	1.09–1.16	< 0.001	1.08	1.04–1.12	< 0.001

CI, Confidence interval; DASS, Depression, Anxiety, and Stress Scale; OR, Odds ratio; SHI, Sleep Hygiene Index. † Adjustment 1: for university degree and exam period. ‡ Adjustment 2: for preceding variables plus DASS_Anxiety, DASS_Stress, DASS_Depression, and/or SHI_total.

**Table 4:** Univariate and multivariate analysis for continuous variable predicting academic performance.

	Crude			Model 1 †			Model 2 ‡		
	Crude r^2^	Crude t	*p*	Adjusted r^2^	Adjusted t	*p*	Adjusted r^2^	Adjusted t	*p*
PSQI total	<0.10	−2.10	0.046	0.15	−1.18	0.238			
PSQI disturbances	<0.10	−2.27	0.024	0.15	−1.58	0.114			
PSQI efficiency	<0.10	−3.12	0.002	0.16	−2.26	0.023	0.16	−1.71	0.087
PSQI latency	<0.10	−2.08	0.038	0.15	−1.21	0.228			
SHI_total	<0.10	−3.90	< 0.001	0.15	−1.59	0.113			
ISI	<0.10	−3.27	0.001	0.16	−2.24	0.026	0.16	−1.09	0.275
DASS_depression	<0.10	−4.02	< 0.001	0.16	−2.26	0.024	0.16	−1.42	0.154
DASS_Anxiety	<0.10	−2.31	0.020	0.15	−0.81	0.416			
DASS_Stress	<0.10	−1.98	0.048	0.15	−0.55	0.580			

BMI, body mass index; DASS, Depression, Anxiety, and Stress Scale; ISI, Insomnia Severity Index; PSQI, Pittsburgh Sleep Quality Index; SHI, Sleep Hygiene Index. † Adjustment 1: for sex, age, BMI, degrees, exam period, physical activity, tobacco, alcohol. ‡ Adjustment 2: for preceding variables plus significant variable in model 1.

## Data Availability

G.P. and L.F.-P. have full access to the data and are the guarantors of the data.
